# P22 *Mycobacterium bovis* intravesical bacillus Calmette-Guérin (BCG)-induced bilateral ankle septic arthritis: a rare late complication of intravesical BCG therapy for bladder cancer

**DOI:** 10.1093/rap/rkae117.053

**Published:** 2024-11-05

**Authors:** Abdulrahman Babiker, Audrey Low

**Affiliations:** Salford Royal Hospital - Northern Care Alliance NHS Foundation Trust, Manchester, United Kingdom; Salford Royal Hospital - Northern Care Alliance NHS Foundation Trust, Manchester, United Kingdom

## Abstract

**Introduction:**

Intravesical bacillus Calmette-Guérin (BCG) therapy is a standard treatment for invasive bladder cancer. While generally safe, it can rarely lead to systemic complications. We present a case of bilateral ankle septic arthritis caused by *Mycobacterium bovis* BCG, manifesting as a rare late complication seven years after intravesical BCG therapy for bladder cancer. This case highlights the importance of considering mycobacterial infection in the differential diagnosis of persistent joint inflammation, especially in patients with a history of BCG therapy. It also underscores the challenges in diagnosis and management of such rare complications.

**Case description:**

A 79-year-old man presented with a 4–5 month history of bilateral ankle pain and swelling in June 2021. His medical history included bladder cancer treated with radical cystoprostatectomy and intravesical BCG therapy in 2016, ischaemic heart disease and osteoporosis.

Initial examination revealed bilateral ankle swelling with warmth. X-rays and MRI scans showed severe degenerative changes and erosions in both ankles, particularly the subtalar joints. Blood tests revealed negative rheumatoid factor and anti-CCP antibodies. Synovial fluid analysis from the left ankle showed an inflammatory pattern.

Despite initial treatment with intra-articular steroids and methotrexate for suspected primary seronegative inflammatory arthritis, the patient’s condition persisted. A large cystic swelling developed on the left ankle, prompting further investigation. Synovial biopsy was performed, revealing granulation tissue with non-necrotising granulomas. Microbiological analysis identified *Mycobacterium bovis* BCG.

The patient was diagnosed with tuberculous arthritis caused by *Mycobacterium bovis* BCG, likely a rare late complication of his previous intravesical BCG therapy. Treatment was initiated with a regimen of ethambutol, rifampicin, and isoniazid (resistant to pyrazinamide). The right ankle subsequently showed similar MRI findings, suggesting bilateral involvement.

After seven months of anti-tuberculosis treatment, the patient continued to experience significant pain and mobility limitations. A chronic ulcer developed at the biopsy site, complicating the clinical picture. The treatment course was extended to 12 months due to the persistence of symptoms and radiological findings.

This case highlights the importance of considering atypical infections in patients who do not respond to initial standard DMARD treatment and to raise awareness that BCG tuberculous arthritis can present years after initial BCG treatment.

**Discussion:**

This case presents a rare and challenging complication of intravesical BCG therapy: bilateral ankle arthritis caused by *Mycobacterium bovis* BCG. The delayed onset, occurring seven years post-treatment, is particularly noteworthy and underscores the importance of maintaining a high index of suspicion for BCG-related complications even years after therapy.

The initial presentation mimicked inflammatory arthritis, leading to misdiagnosis and delay in appropriate treatment. This highlights the need for clinicians to consider atypical infections, especially mycobacterial, in patients with a history of BCG exposure presenting with persistent joint inflammation.

Diagnosis was complicated by the insidious onset, bilateral involvement, and the absence of systemic symptoms typically associated with tuberculosis. The case emphasises the value of tissue biopsy and extended cultures in reaching a definitive diagnosis in complex cases of arthritis.

Treatment of *Mycobacterium bovis* BCG arthritis presents unique challenges. The organism’s inherent resistance to pyrazinamide necessitates a modified anti-tuberculosis regimen. The prolonged course of antibiotics (extended to 12 months in this case) reflects the difficulty in eradicating the infection, particularly in a joint space.

The development of a chronic ulcer at the biopsy site and the potential involvement of the contralateral ankle further complicate management. This case raises important questions about the optimal duration of therapy and the role of surgical intervention in such cases.

Long-term sequelae, including chronic pain and mobility issues, highlight the potential for significant morbidity associated with this rare complication. This case underscores the need for a multidisciplinary approach involving rheumatology, orthopaedics, infectious diseases, and pain management in treating such complex cases.

**Key learning points:**

• **Late-onset complications:** Intravesical BCG therapy can lead to systemic complications, including arthritis, even years after treatment. Clinicians should maintain a high index of suspicion in patients with a history of BCG exposure presenting with persistent joint symptoms.

• **Diagnostic challenges**: BCG-induced arthritis can mimic other forms of inflammatory arthritis, leading to potential misdiagnosis. A thorough history, including past BCG therapy, is crucial in formulating a differential diagnosis.

• **Importance of tissue diagnosis:** In cases of atypical or treatment-resistant arthritis, synovial biopsy with extended cultures for mycobacteria can be pivotal in reaching a definitive diagnosis.

• **Treatment considerations:** *Mycobacterium bovis* BCG is inherently resistant to pyrazinamide, necessitating a modified anti-tuberculosis regimen. Extended treatment courses may be required, and close monitoring for response is essential.

• **Multidisciplinary approach:** Management of BCG-induced arthritis requires collaboration between rheumatology, orthopaedics, infectious diseases, and pain management specialists to address the complex interplay of infection, joint destruction, and chronic pain.

• **Long-term sequelae:** BCG-induced arthritis can lead to significant joint destruction and chronic pain, potentially resulting in long-term mobility issues and decreased quality of life.

• **Surgical considerations:** The role of surgery in BCG-induced arthritis is complex, with potential risks of exacerbating infection or poor wound healing. Decisions regarding surgical intervention should be carefully weighed against conservative management.

• **Patient education:** Patients receiving intravesical BCG should be informed about the potential for rare, late-onset complications and advised to report persistent joint symptoms, even years after treatment.

• **Research implications:** This case highlights the need for further research into the pathogenesis, optimal treatment duration, and long-term outcomes of BCG-induced arthritis to improve management strategies for this rare but significant complication.

